# Long-Term Homeostatic Properties Complementary to Hebbian Rules in CuPc-Based Multifunctional Memristor

**DOI:** 10.1038/srep35273

**Published:** 2016-10-20

**Authors:** Laiyuan Wang, Zhiyong Wang, Jinyi Lin, Jie Yang, Linghai Xie, Mingdong Yi, Wen Li, Haifeng Ling, Changjin Ou, Wei Huang

**Affiliations:** 1 Key Laboratory for Organic Electronics and Information Displays & Institute of Advanced Materials (IAM), Jiangsu National Synergetic Innovation Center for Advanced Materials (SICAM), Nanjing University of Posts & Telecommunications (NUPT), 9 Wenyuan Road, Nanjing 210023, China; 2 Key Laboratory of Flexible Electronics (KLOFE) & Institute of Advanced Materials (IAM), Jiangsu National Synergetic Innovation Center for Advanced Materials (SICAM), Nanjing Tech University (NanjingTech), 30 South Puzhu Road, Nanjing 211816, China

## Abstract

Most simulations of neuroplasticity in memristors, which are potentially used to develop artificial synapses, are confined to the basic biological Hebbian rules. However, the simplex rules potentially can induce excessive excitation/inhibition, even collapse of neural activities, because they neglect the properties of long-term homeostasis involved in the frameworks of realistic neural networks. Here, we develop organic CuPc-based memristors of which excitatory and inhibitory conductivities can implement both Hebbian rules and homeostatic plasticity, complementary to Hebbian patterns and conductive to the long-term homeostasis. In another adaptive situation for homeostasis, in thicker samples, the overall excitement under periodic moderate stimuli tends to decrease and be recovered under intense inputs. Interestingly, the prototypes can be equipped with bio-inspired habituation and sensitization functions outperforming the conventional simplified algorithms. They mutually regulate each other to obtain the homeostasis. Therefore, we develop a novel versatile memristor with advanced synaptic homeostasis for comprehensive neural functions.

The development of electronics enlightens us that, for a fundamental element, the extension of each additional function may have considerable advance for the integrated electronic chip, whereas the neglect of a certain property may induce the ineffectiveness of upper-level chip functions. With simple structure which enable functional scaling well beyond CMOS-based circuits and inherent parallel information processing and storage, the memristor is becoming an excellent choice to design the specific artificial synapse for developing neuromorphic chips^1^,^2^,^3^,^4^,^5^,^6^,^7^. Based on the memristive characteristics, namely, operation history-dependent tunable and storable conductance, the novel interdisciplinary ideas are proposed whereby electrical stimuli represent neural spikes and resultant current corresponds to the synaptic weight^1^,^2^. And then, indeed, significant learning and memory processes based on the adjustable neuroplasticity in frameworks of the fundamental Hebbian rules and the revised version spike-timing-dependent plasticity (STDP) rules have been implemented phenomenologically through the simple devices^1^,^3^,^5^,^6^,^8^,^9^,^10^,^11^,^12^,^13^,^14^. However, the prevalently simulated rules developed for artificial neural networks (ANNs) are simplified rules that miss out the homeostatic property of neural activities presented in the realistic neural networks^15^,^16^,^17^,^18^,^19^,^20^, outperforming ANNs. It is desirable to endow the homeostatic property with the emulated neural activities in memristors to enhance the ability of self-stabilizing.

Biologically, excitatory and inhibitory activities are fundamental processes of synaptic activities. They mutually restrict and coordinate each other to form diverse complex activities under alternant stimuli. The Hebbian rules, which can be summarized as “neurons that fire together, wire together”, focus on simplex synaptic activities and can hardly ensure the long-term activities immune to be excessively excitatory or inhibitory^15^,^18^,^19^,^21^, which potentially may lead to the collapse of neural networks. The homeostatic mechanisms are necessary to balance neural activities and ensure stability over long time both in the realistic neural networks and ANNs, and they are attracted considerable attentions in the fields of biological realistic neural networks and electronic ANNs. However, in the artificial synapses, few results investigate the property of long-term reciprocal regulation of two elementary activities for specific homeostatic states to date. As reported in prevalent devices, a balance of the conductive states under coordinated excitatory and inhibitory stimuli can be maintained^1^,^12^,^22^. However, the equilibrium can be broken under unbalanced signals, and a new balance cannot be established^23^,^24^, thereby violating the key biological principle—homeostatic plasticity^17^,^18^,^25^,^26^, aiming at ensuring the synaptic dynamic balance complementary to Hebbian rules. As one advanced adaptive mechanism, it has been applied to design versatile materials effectively^27^. Therefore, in the electronics, it is necessary to create an memristive element with homeostatic plasticity to enhance the long-term stability of the overall conductivity under excessive stimuli through self-adjustment^28^,^29^. In another situation for the long-term homeostasis of neural activities, during learning processes the overall response in our brain is gradually weakened under repeated moderate signals and recovered by intense signals, termed as habituation and dishabituation (sensitization) which are also neglected by Hebbian rules^30^. They regulate each other to contribute to achieving the neural homeostasis. Otherwise, failed habituation/sensitization may lead to the exorbitantly high/low level of neural activities. Considered as two efficient non-associative learning modes commonly addressed by biologists^31^,^32^,^33^, they can help us selectively respond to external signals, namely, by paying less attention to unimportant stimuli and more attention to novel stimuli^34^. And habituation has been emulated through software^35^,^36^. It is a pity, however, that their systematically inter-regulatory properties have not been detailedly implemented in a single memristive platform. In addition, from the perspective of device design and construction, the prevalent physical microprocesses for developing memristors are mainly based on shaping of the ion-type conductive filament under electric field^3^,^9^,^12^,^22^,^24^,^37^,^38^,^39^, of which the smoothness and gradient of conductive state are highly sensitive to the layer composition and the bulk and interface components. Thus, it may be awkward to precisely control the growth location, orientation and rate of ionic filaments for inner imperfect structures in these devices^39^,^40^.

In this paper, we construct the memristor using the organic semiconductor copper phthalocyanine (CuPc) based on the space-charge-limited conduction (SCLC) transition influenced by different degrees of trapping charges as a function of the operation history. This non-filamentary memristive transition is induced by the reversible charge trapping and detrapping processes. Consequently, impressive memristive characteristics, including distinguishable smooth ascending/degressive I–V hysteresis and gradually changing intermediate states, are demonstrated under sequent scans. Besides the emulations in accordance with the Hebbian rules, the CuPc prototype can emulate the homeostatic plasticity. Not only is the balance maintained under coordinated stimuli but also can the initial imbalance under uncoordinated stimuli be repaired, and a new balance is established after self-adjustment. In addition, different from this homeostasis pattern, in thicker samples, the changing tendency of the overall excitatory conductivity is strongly dependent on the stimulus amplitude, which tends to decrease under modest stimuli, resembling neural habituation, but can be recovered under stronger spikes, as dishabituation implies^41^. These inter-regulatory mechanisms are also emulated for the long-term homeostasis of neural activities.

## Results

### Memristive behaviours in CuPc-based samples

**Figure 1:**
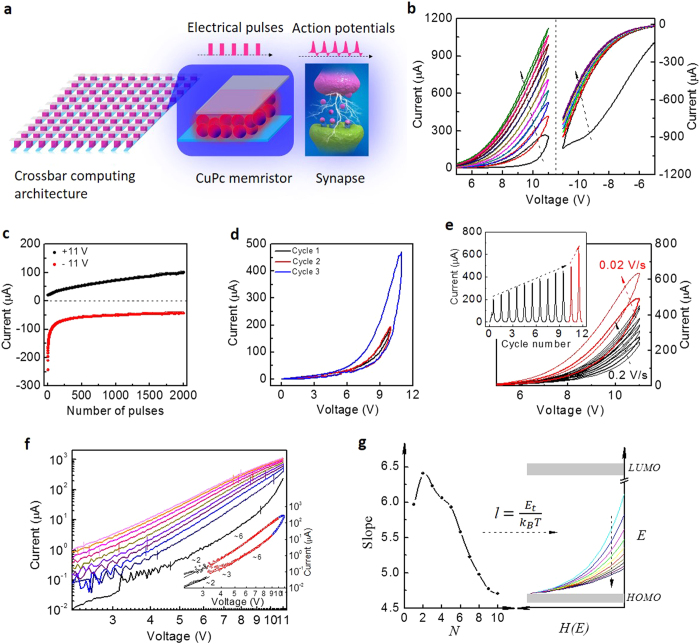
Tunable conductivity under cyclic sweeps in the ITO/CuPc/Al memristor. (**a**) Schematic diagram of the CuPc-based crossbar architecture and one typical CuPc element compared with a biological synapse on the right. The applied electrical inputs correspond to the synaptic action potentials. The thickness of the evaporated CuPc film is monitored by a quartz crystal cymometer Δ*f* = 800 Hz (~50 nm). Device size: 100 × 100 μm^2^. (**b**) Memristive current under 10 cyclic voltage sweeps (0 → 11/−11 V → 0). (**c**) Polarity-dependent EPIR effect. The current progressively increases/decreases under a series of electrical pulses. (**d**) Tuning current curves with different sweeping amplitudes. The sweeping amplitude of the first two cycles is 10 V, and the third one is 11 V. (**e**) Current hysteresis curves at different scanning speeds, 0.2 V/s of the first 10 cycles and 0.02 V/s of the last two cycles. Inset shows the current tendency versus the cycle number. The increasing stride at the maximum voltage increases by ~5 times as the scanning rate is reduced tenfold. (**f**) Log-log plots of the forward scanning current extracted from (**b**). Inset shows the fitting slope of the first cyclic current curve. Backward curves nearly coincide with the following forward curves. Fitting results demonstrate that the current is trap-affected SCLC, and there exists a dominant SCLC region in each sweep (undergoing the maximum range) marked by the short bars. (**g**) Schematic diagram of the correlation between decreasing slopes of dominant SCLC during 10 forward sweeps in (**f)** and the gradual variation of exponentially distributed state density. More and more traps are filled by retentive charges, causing gradually reducing trap sites which can be measured by the integral area under each exponential curve.

The crossbar structure for neuromorphic computing are prepared by evaporating the CuPc layer and top Al electrodes successively in a vacuum onto a glass substrate with bottom ITO electrodes. There is no need to precisely control the stoichiometric ratio of the active layer compound. It thus appears that the processing is relatively simple and inexpensive^42^. Fig. 1a shows the schematic image of ITO/CuPc/Al memristor. The memristive behaviours of enhancing and suppressing hysteresis loops energized by 10 cyclic positive and negative voltage sweeps are presented in Fig. 1b. The electrical signals are applied to ITO electrode and Al electrode is grounded. Ten clearly distinguishable positive intermediate states are obtained corresponding to our short-term-memory capacity (5~9) in terms of the Miller’s Law^43^. Notably, the CuPc-based memristor shows smooth adjacent I-V characteristics without saltation or fluctuation during the transitions of conductive states, implying the quasi-continuous distribution of conductivity in the organic artificial synapse. Corresponding to the sweeping memristive behaviour, the pulse polarity-dependent electric-pulse-induced resistance (EPIR) effect has attracted extensive interest as an effective mechanism for memory devices. The EPIR effect is primarily investigated in the inorganic ReAMO systems (Re, rare-earth ions; A, alkaline ions; M, transition-metal ions; O, oxide)^44^,^45^, and it is also experimentally demonstrated in CuPc memristors as shown in Fig. 1c. The EPIR responses are considered as the basic excitatory and inhibitory activities to investigate the complex stimulus-response rules for synaptic imitations.

The physical mechanisms for memristive behaviours can be mainly attributed to the charge trapping/detrapping processes with different filling ratios and energy levels within CuPc micro-domains^46^,^47^. In the CuPc sandwich structure, we consider a case of hole injection, transport and trapping. In the organic semiconductor polycrystalline samples as indicated in Fig. S1, the CuPc domains serve as charge reservoirs and their interfacial regions containing a large number of traps to impede current serve as barriers^46^,^47^. The current is given by the carrier hopping processes travelling from one domain to another, and the resistance arises from intergranular and interfacial impedances. The increasing concentration of carriers captured at the trapping sites causes their stronger correlation^46^, namely, the occurrence of more overlaps of carrier wave functions and abduction of Mott transition^47^,^48^,^49^,^50^. Thus, higher conductive percolation channels in insulating regions between CuPc domains are formed as a function of the trapping carrier concentration^46^. As a result, the current continuously increases relying on the preceding accumulated operation, implying the typical memristive property.

To gain insight into the mechanisms, the transport pattern of the carriers is further investigated. As shown in Fig. 1f, the fitting results of the typical first cycle demonstrate that the current is trap-affected SCLC during the large sweeping ranges. And the same is true for the rest cycles. The SCLC can be subdivided into two types, SCLC affected by discrete traps with shallow energy levels (given by *J* ∝ *θU*^2^/*d*^3^) and SCLC affected by exponential distribution traps with deep energy levels (given by *J* ∝ *U*^*l*+1^/*d*^2*l*+1^, *l* > 1). Considered as a criterion to characterize the distribution width of trapping charges, *l* can be written as


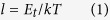


where *E*_*t*_ is the characteristic energy level of exponentially distributing trapping charges. And the state density *H*(*E*) as a function of the energy level *E* decreases as *E* increases, which can be expressed as


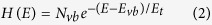


where *E*_*vb*_ and *N*_*vb*_ are the valence bond energy level and state density at the valence bond edge, respectively^47^.

As shown in the inset of Fig. 1f, the current changes from the low-exponent SCLC affected by shallow traps (slope~2) to the high-exponent SCLC affected by deep traps (slope >6) as the voltage increases. More injected carriers fulfill the trapping sites in order of deeper energy levels. Some of these carriers are released and others can be stored into deep sites as the voltage decreases. It is harder for those in deep trapping sites to escape, thereby resulting in the presence of stable hysteresis after a cyclic scanning (Fig. 1b)^46^,^47^. As evidences of memorized trapping charges, a large enough voltage is necessary for sufficient charges to occupy deep traps, while low voltage sweeps cannot result in the retentive trapping charges as demonstrate in Fig. 1d. Furthermore, both the lobe area and increasing current stride expand monotonically as the scanning pace decreases (Fig. 1e), as typical fingerprints of the memristor^7^,^51^, which can be attributed to the improvement of total flowing charges in one slow period. During following cycles in Fig. 1b, the forward increasing current closely clings to the previous curve and results in more retentive trapping charges. Thus new hysteresis loops appear consecutively. Meanwhile, the trap exponent *l* of the 10 cyclic main SCLC gradually decreases for decreasing trapping sites to affect SCLC. In conclusion, higher concentration of retentive trapping charges results in the stronger charge correlation and creates more highly conductive percolations. The conductance gradually approaches the saturation during the following scans, and the hysteresis area diminishes continuously because the number of trapping sites gradually reaches the saturation state. The distribution of trapping charges to affect SCLC is narrowed, as clearly demonstrated by the decreasing slope of the forward dominant SCLC illustrated in Fig. 1f,g. In the opposite case, the retentive trapping charges are progressively released (or neutralized by reverse charges from another angle) under the reverse voltage, which results in the decreasing conductive state. And at the initial stage, the deceasing rate is much higher for the rapid neutralization process of the relatively sufficient trapping holes. In the complex hopping systems, the relaxed current can be modelled by the stretched exponential equation


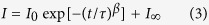


where *τ* is the time constant, *I*_0_ is the initial level of the decay current, *I*_∞_ is the final value and *β* is the stretch index ranging between 0 and 1, known as the Kohlrausch law^14^,^21^,^52^. Compared with the simplified exponential equation^9^,^12^, two parameters, *τ* and *β*, in the Kohlrausch curve can set up in-depth communication between the description of the mimetic memorization events and the internal microscopic physical adjustment^52^.

### Simulations of Hebbian rules and homeostatic plasticity

From the fundamental to the complex neural activities, the potential to imitate the neuroplasticity in a simple prototype compared with the complex CMOS-based neuromorphic chips, is the crucial reason why the memristor is regarded as a novel basic element. The characteristics that distinguish the memristor from general electronic devices (resistors, capacitors and inductors) are their input-dependent device states and variations, as defined in the frameworks of


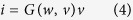



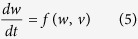


where *w* is the state variable of the device. Resembling the synaptic weight of a synapse, the conductive state in a memristor reflects the efficiency for signal transmission, which can be shaped according to external accumulative activations for specific neural computing^2^. Actually, the input amplitude, duration and frequency-dependent variations related to the essential learning/memory laws are widely emulated for synaptic computation^1^,^3^,^5^,^6^,^8^,^9^,^10^,^11^,^12^,^13^,^53^.

**Figure 2:**
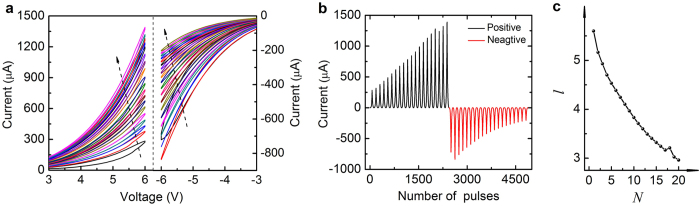
Tunable conductive states of the ITO/MoO_3_/CuPc/Al memristor. (**a**) 20 cyclic I-V characteristics under consecutive positive/negative voltage sweeps (0 → 6/−6 V → 0). (**b**) The trend in the current versus pulse number for the data shown in (**a**). (**c**) The decreasing slopes of the dominant SCLC during 20 positive forward sweeps in ITO/MoO_3_/CuPc/Al memristor.

In the CuPc-based memristor, after preparing a buffer layer MoO_3_ (several nanometers) between ITO and CuPc layer, the operation voltage for memristive behaviours can be reduced because the MoO_3_ layer can reduce the energy barrier for hole injection effectively as demonstrated in Fig. 2a. The conductive behaviours of carriers are also trap-affected SCLC during the most sweeping ranges and Fig. 2c shows the decreasing slope of the dominant SCLC versus cycle number. Moreover, as shown in Fig. S2 the current levels of the samples linearly scale with the device area, and the changing percentage of the current states is independent on the area, which indicate the homogeneous memristive mechanism. And the current density in this organic sample is lower than other samples^3^,^8^,^12^,^13^. Both of them are conductive to reduce the energy consumption. The impressive memristive behaviours in ITO/MoO_3_/CuPc/Al, with reliably smooth characteristics and multiple intermediate states for improving the modulatory space, provide us a fine prototype to simulate the neural functions. Positive stimuli that lead to increasing conductance represent excitatory actions, and the following negative stimuli represent inhibitions. Achievement of the input-dependent enhancement of synaptic weight is of popular interest, such as post-tetanic-potentiation (PTP), short-term memory (STM) and long-term- memory (LTM) which are also effectively implemented in CuPc-based artificial synapse as shown in Figs S3 and 4. These bio-inspired functions are based on the fundamental Hebbian rules, which imply that stronger accumulative activations result in stronger responses.

**Figure 3:**
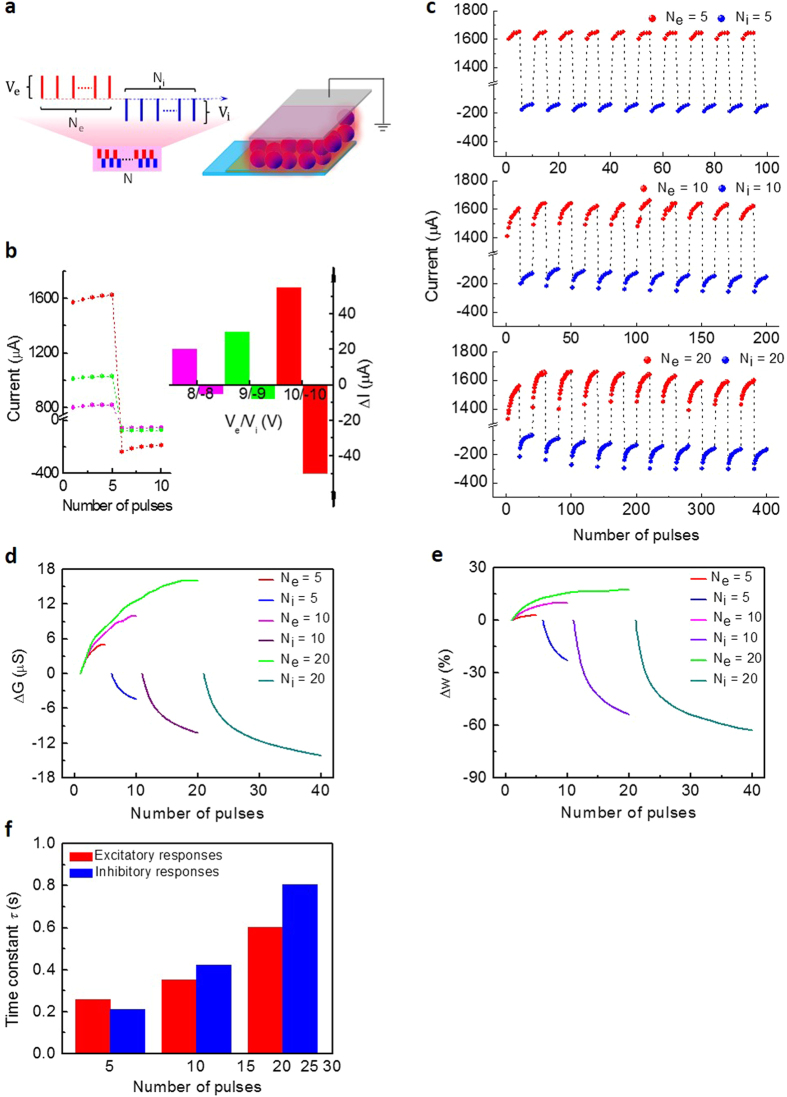
Balanced memristive responses under coordinate stimuli. (**a**) Schematic diagram of alternant excitatory and inhibitory inputting pulses applied to the ITO/MoO_3_/CuPc/Al device consisting of different number of positive/negative pulses N_e_/N_i_ in each period. *N* is the number of applied periods. **(b)** The increasing/decreasing current under positive/negative pulses (V_e_/V_i_ = 8/−8 V, 9/−9 V, 10/−10 V and N_e_/N_i_ = 5). The right diagram shows the ultimate increment/decrement in different amplitude modes. The variations in low-voltage modes are unobvious. **(c)** The periodic increasing/decreasing current under the same periods of positive/negative pulses in three modes with different N_e_/N_i_ (V_e_V_i_ = 10/−10 V, N = 10). (**d**) The variations of the conductance (ΔG) (defined as ΔI/V) versus N_e_/N_i_ in the typical periods of Fig. (**c)**. (**e**) The corresponding synaptic weight (Δw) (defined as ΔG/G). (**f** ) Histogram of the time constants τ (calculated from the stretched exponential equation) of excitatory and inhibitory currents extracted from (**e)**, and increasing 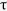 means that more time is required to reach the respective saturated values in different modes.

**Figure 4:**
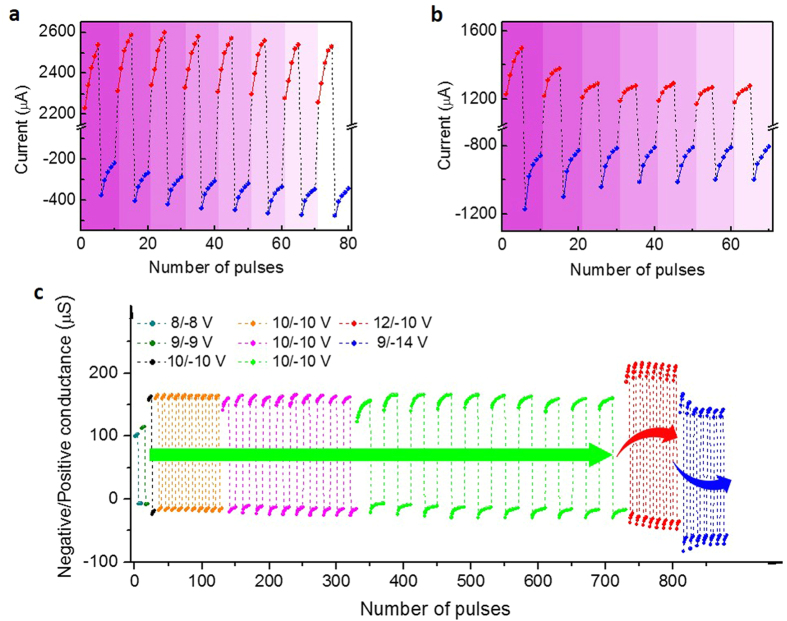
Homeostatic memristive responses under non-coordinated pulses. (**a,b**) The current gradually increases/decreases under pulses of V_e_/V_i_ = 12/−10 V **a** and 9/−14 V **b**. (**a**) Compared with the mode of V_e_/V_i_ = 10/−10 V, the positive current grows stronger, while the overall negative level is gradually enhanced till they reach a homeostatic state as more periods are applied. (**b**) Greater non-equilibrium inputs enlarge the initial unbalanced states. The greater negative overall current gradually relaxes, while the overall positive level is gradually depressed till they reach a new stable balanced state. (**c**) The comprehensive comparisons of the overall positive and negative conductance and long-term regulatory changes in different V_e_/V_i_ and N_e_/N_i_ modes extracted from Figs 3b,c and 4a,b, respectively.

**Figure 5:**
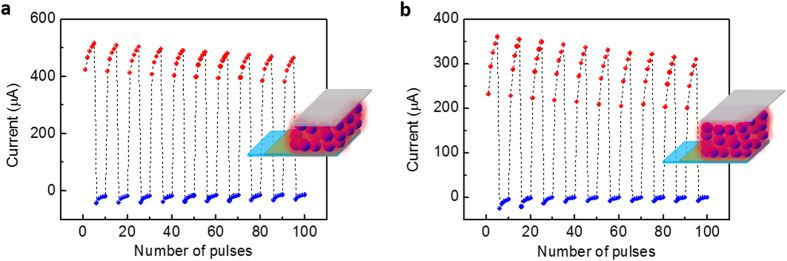
Tunable conductive states in thicker CuPc-based memristors. (**a,b**) Gradually relaxing overall current in thicker memristors (Δf = 1600 Hz ~70 nm of (**a**) and 3200 Hz ~90 nm of (**b**), (N_e_/N_i_ = 5, V_e_/V_i_ = 10/−10 V, and N = 10). The current progressively increases/decreases under positive/negative pulses in each period, whereas the overall current gradually decreases as more periods are applied, and the relaxation trend is more obvious in (**b**) than in (**a**). Insets display schematic diagrams of thicker memristors (1600 Hz (**a**) 3200 Hz (**b**).

**Figure 6:**
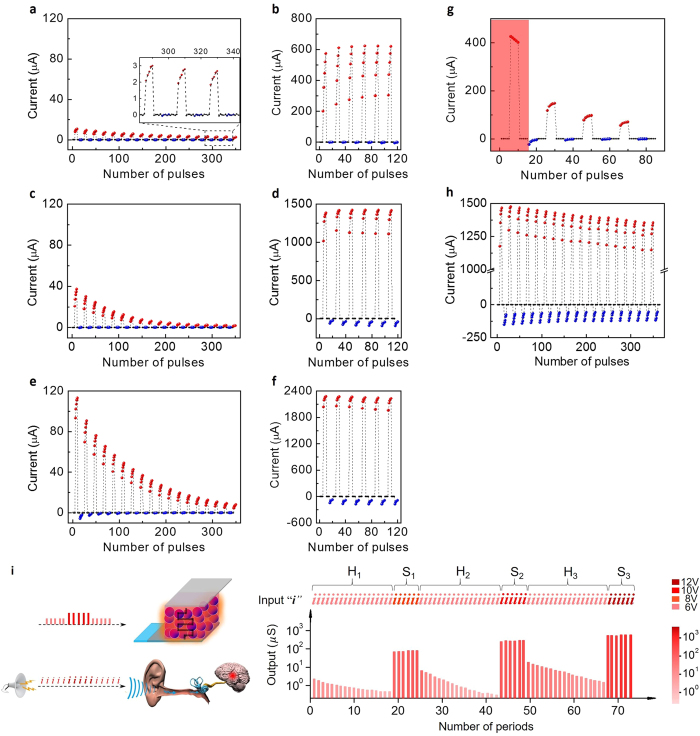
Habituation and sensitization behaviours. (**a**–**f**) As periodically alternant stimuli are applied, the current gradually increases/decreases under 5 pulses of 6/−6 V (**a**,**c**,**e**,**g**), 8/−8 V (**b**), 10/−10 V (**d**) 12/−12 V (**f** ) in an ITO/MoO_3_/CuPc (3200Hz)/Al memristor. The applied stimuli are further illustrated in Fig. S9b. (**a**) The overall average current gradually decreases under modest inputs. However, the excitatory level is gradually enhanced under strong stimuli (**b**) and the current after being motivated by sensitive pulses is higher than before (**c)**. The stronger sensitive pulses are (**b**,**d**,**f** ) the higher excitatory levels are reached under both sensitive and following habitual actions (**a**,**c**,**e**,**g**). The sequential habituation and sensitization in (**a**–**f** ) are labelled as H_1_, S_1_, H_2_, S_2_, H_3_ and S_3_ stages, respectively. (**g**) Refractory period. After applying intense pulses (**f** ) the current cannot be immediately excited by the first 5 modest pulses (red region). (**h**) The average excitatory current under intense pulses slowly relaxes as more periods are applied (V_e_/V_i_ = 10/−10 V). (**i**) Graphical auditory responses of habituation and sensitization in H_1_, S_1_, H_2_, S_2_, H_3_, and S_3_ stages extracted from (**a**–**f** ). Assuming that the periodic habitual and sensitive inputs in different stages with different amplitudes correspond to a series of voices with different intensities entering the auditory system marked by the character “***i***” with different colour depths, and the overall conductance corresponds to the auditory response marked by the red bars with different intensities and depths.

In neural system the long-term inter-regulation of the elementary excitation and inhibition under the condition of ensuring the homeostatic state is the essential behaviour, and it is likewise significant for a neuromorphic device. Here, the self-adjustment for dynamic balance is investigated in detail, which depends on not only the programming protocol but also the device configuration. The corresponding results in different modulatory patterns are shown in Figs 3, 4, 5, 6. Figure 3 shows the balanced responses under coordinate stimuli. The periodic positive and negative stimuli are applied to represent the alternant excitatory and inhibitory stimuli as illustrated in Fig. 3a, which are the familiar stimulating events in our neural activities. The current exponentially increases/decreases under consecutive positive/negative actions as a function of the pulse number, and the total increment/decrement increases in magnitude as V_e_/V_i_/increases (Fig. 3b). Here we set 10 V and −10 V as the excitatory and inhibitory amplitudes. As shown in Fig. 3c, the device conductive state can be reproduced under the coordinated pulse sequences. The dynamic balance of the overall conductive state can be maintained over a long period regardless of the pulse number in each period. Moreover, through an analysis of these different responsive modes, it can be concluded that the responsive curves under more activations are not purely-prolonged tendencies following the less-activated responses. In the modes with larger N_e_/N_i_, the conductive variations and corresponding synaptic weight under the same stimuli number are enhanced as demonstrated in Fig. 3d,e. The results indicate that different responsive modes are developed for improving the response efficiency in larger N_e_/N_i_ modes, thereby embodying the self-adaptive characteristics of CuPc-based artificial synapse. And the activity-dependent multi-mode features are typical properties of neural networks^54^,^55^. The adjustable processes can be observed in detail from the beginning periods in larger N_e_/N_i_ modes in Fig. 3c. Importantly, the exponentially changing current in each stage implies the tendency that the responsive efficiencies (the average variations of conductive states under the same activations) are asymptotically suppressed and reach saturations for gradually saturated trapping charges. This bio-mimetic property in CuPc memristor is in favour of avoiding excessive excitation or inhibition for regulating the long-term homeostasis of neural activities^17^, going beyond the simplex Hebbian rules. In addition, the time constants underneath these increasing and decreasing currents are also tuned with the same trends in accordance with N_e_/N_i_ as summarized in Fig. 3f. It implies that the regulation of excitatory response rate is accompanied by similar regulation of inhibitory activities under the coordinated stimuli. These synchronous regulatory mechanisms are also conducive to the synaptic stability for adapting to changing conditions^12^,^56^.

In addition to performing the fine dynamic stability under balanced inputs, more importantly, the CuPc-based memristor can exhibit homeostatic plasticity under uncoordinated stimuli after a period of self-adjustment. Indeed, in realistic neural networks and ANNs there exist more unbalanced signals. And thus it is necessary for synapses to reasonably regulate the neural activities and maintain their stability. During the initial period in Fig. 4a, the memristor behaves unsteadily as soon as unbalanced pulses are applied, exceeding the overall excitatory conductivity in Fig. 3c. When more unbalanced periods are applied the overall excitatory and inhibitory conductivity is progressively enhanced for stronger excitatory inputs. However, instead of increasing all the time, the overall conductive levels gradually reach a unified steady state after several periods of their inter-coupling processes. Subsequently, the stronger positive pulses do not result in excessive excitation after self-adjusting in the CuPc-based artificial synapse. Notably, when great non-equilibrium pulses are applied as shown in Fig. 4b, the overall states exhibit more unstable behaviours initially than before. The inhibitory affect is gradually enhanced relative to the suppression of excitatory activity for intenser inhibitory inputs. In this case, the overall activities persistently adjust themselves to reach a steady state. The characteristics indicate that CuPc samples can perform homeostatic plasticity effectively under unbalanced stimuli for inner asymptotically saturated trapping sites as discussed above, which are beyond recent results of the filamentary synapse and meet the neural and model requirements of long-term homeostasis^24^,^57^.

Through comparisons of the conductive variations in different modes as analyzed in Fig. 4c, it can be demonstrated that the overall steady conductive states after self-adjusting, as well as the states under coordinate pulses, differ from each other, which embodies the synaptic activity-dependent multiple-mode characteristics in CuPc samples. That is to say, the different adaptive modes and steady states of network activity can be obtained according to the different stimulus situations.

Furthermore, under the testing conditions of the same excitatory/inhibitory bias (V_e_/V_i_), much more pulses in each excitation/inhibition stage (N_e_/N_i_ = 40, 80) are also investigated as shown in Figs S5 and S6. The results reveal that the overall current can still be balanced under the coordinated signals (V_e_/V_i_ = 10/−10 V) as shown in Fig. S5a,b. And under uncoordinated stimuli (V_e_/V_i_ = 12/−10 V in Fig. S6a,b, V_e_/V_i_ = 9/−14 V in Fig. S6c,d), the overall conductive state can perform asymptotic stability after a period of self-adjustment. Although the pulse number is greatly enlarged, the overall current levels do not keep increasing/decreasing in the modes with stronger excitatory/inhibitory inputs. In addition, the enlarged pulse number in each period implies that the differences between the excitatory and inhibitory stimulations are enhanced, which result in the expanded ranges of the dynamically changing neuroplasticity and the amplified variations of the overall activities relative to the initial states of CuPc memristors as indicated in Fig. S7a,b.

### Simulations of habituation and sensitization for homeostasis in thicker samples

In additon to focusing on implementing specific programming parameters for fresh learning rules, the internal structural cause is also carefully modified. Recent studies have demonstrated that the synaptic weight and time constant of signal transmission are configuration-dependent^22^. Herein, considering the crucial role of the CuPc layer in carrier transporting and trapping, the memristive characteristics for specific synaptic emulations in thicker samples are explored. Except for the analogous sweeping memristive behaviours in the thin sample, the thick memristors show lower conductive states and enhanced rectifying properties (Fig. S8a–d). As expected, the synaptic weight can be enhanced/suppressed as soon as serial excitatory/inhibitory spikes are applied in each period as demonstrated in Fig. 5a,b. Unlike the foregoing thin sample, the overall excitatory conductance under periodic coordinated stimuli tends to be weakened progressively. From the perspective of bio-inspired computing, it enlightens us that the gradual suppression of the overall excitation is analogous to the biological habituation. From the electronic version, biological habituation and sensitization are unfamiliar simulations, which are ubiquitous events of our neural activities. In the nervous system, habituation tends to suppress our overall responses under modest signals, contrary to the sensitive performance tending to re-enhance the responses through intense stimuli. During our long-term neural activities, they regulate each other to achieve the homeostatic state. Otherwise, failed habituation/sensitization may lead to the exorbitantly high/low level neural activities. These mechanisms are also missing in Hebbian rules in addition to the homeostatic plasticity^19^,^30^,^34^. Although the relevant accurate algorithms of the inter-regulatory mechanisms have not been developed, the plasticity can be implemented in thicker CuPc memristors. For a synapse, the ability to dynamically remodel its response mode in accordance with external variable stimuli for appropriately self-adjusting its weight and memory is the key capacity, termed as the activity-dependent property. And it is likewise for the neuromorphic device. The activity-dependent performance (habituation and sensitization) is different from the general endurance issue in a memristor. It is noteworthy that the gradual relaxation of habituation refers to long-term adjustable behaviours under periods of modest irritations, which is not in conflict with the excitatory enhancement under transient high-frequency stimuli. The initial temporary excitation is necessary for neural activation under habitual stimuli, however there is no obvious excitation in thin samples under modest voltage as shown in Fig. 3b.

Biologically, Kandel *et al.*^31^ performed systematic studies on exploring biological habituation and sensitization. In their experiments, a neuron can be excited under transient electrical impulses, and the overall excited amplitudes of the EPSPs gradually decrease under 15 input stages with moderate intensity and long intervals of 10–60 s, where the resting time is much longer than the activating time, which is necessary to silence the exciting synapse for avoiding the interferences of PTP performance under frequent inputs^6^,^9^,^14^. In the CuPc artificial synapse, considering an effective method for imitations based on relevant biological operation, the insertion of a serials of negative pulses to promote conductive recovery is devised following excitation, which is a substitutive operation for the long-term relaxation period (Fig. S9a).

As shown in Fig. 6a, the overall excitatory trends under alternate modest stimuli gradually decrease, implying that it is increasingly difficult to activate the artificial synapse as more periods are applied, resembling the neural habituation. The habituation of current levels can be removed after placement for several hours as shown in Fig. S10, resembling the dishabituation as time goes on. Importantly, they exhibit different responsive patterns according to the signal intensity. As stronger stimuli are applied, the suppressed excitatory levels gradually increase as shown in Fig. 6b. Furthermore, the suppressed conductive states under moderate pulses are also enhanced by the sensitive activations as demonstrated in Fig. 6c, in analogy with the synaptic behaviour of sensitization for intense stimuli. In fact, sensitization is often characterized through a universal responsive enhancement under the whole range of stimuli, including strong and modest ones. After removing sensitive stimuli, habituation still occurs under habitual inputs as expressed in Fig. 6c. From the perspective of long-term homeostatic state, the habituation and sensitization mechanisms tending to suppress and enhance the synaptic responses, respectively, regulate each other to achieve the dynamic balance state in case of exorbitantly high/low-level neural activities. Moreover, the stronger sensitive inputs are (Fig. 6d,f), the higher excitatory levels can be reached under sensitive pulses, and likewise for the following habitual pulses (Fig. 6c,e,g). As the sensitization implies, stronger stimuli result in the progressive amplification of our comprehensive response. Notably, for the sensitized high conductive state, there is subsequent habituation, although sluggishly (Fig. 6h), which conforms to the universal property of biological habituation and demonstrates the inherent property of suppression in thicker samples^58^. Therefore, we can conclude that the self-adaption for dynamic balance observed in neurons can be emulated effectively by the thicker CuPc memristors. The coupled performances can help us selectively pay less attention to weak signals and more attention to intense signals as mentioned above. As an intuitive expression in our sensory neural system, we can utilize the advanced electrical performances in a single thicker sample to implement the habituation and sensitization in the auditory system, as detailedly illustrated in Fig. 6i. This demonstrates that the single element can effectively function as a hardware sensory system to generate the brain-like pattern of habituation and sensitization. In the thicker samples, it is more difficult to inject carriers, especially electrons, as indicated by the lower conductive state and the enhanced rectification, and thus there would exist abundant space charges accumulating at the electrode interfaces to fill interfacial traps with different energy levels. The current characteristics including saturated processes (transition from trap-filled SCLC slope 2 to trap-saturated SCLC slope = 2 in Fig. S8e,f) implies the sufficient space charge accumulations^47^. Meanwhile, the large amount of electrons accumulated at the interfaces would enhance the hole neutralizing processes. As a result, when negative pulses are applied followed by positive pulses, except for a portion to neutralize trapping holes, there are additional negative charges accumulating at the interfaces. Therefore, when the next periodic positive pulses are applied, the existing reverse charges weaken the overall conductivity compared with the former period, and similar patterns likewise occur as subsequent periodic negative pulses are applied. Consequently, the overall excitatory level progressively decreases. And the injecting capacity of carriers can be enhanced under high voltage. However, the abundant interfacial charges accumulated in the thicker samples still cause sluggish habituation.

In addition to the long-term self-adaptive performance, the simplified Hebbian algorithms are also inadequate for describing the temporary sophisticated phenomena compared with realistic neural networks; one typical example is the refractory period. Biologically, there exists an interval (several ms) during which a neuron excitedly triggered by preceding spikes is incapable of being excited again by weak actions^58^,^59^. The existence of a refractory period is an essential biological property for self-preservation. Interestingly, there is electronic refractory period in the CuPc-based artificial synapse where the electrical performance is superior to that of Hebbian rules. The current progressively increases under strong excitatory pulses and then rapidly decays once the activations are removed, and the decreasing trend cannot be reversed by closely following weaker pulses (Fig. 6g)^59^. From another perspective, the incapability under weaker actions can be ascribed to the increasing threshold voltage for motivating the exciting neurons^60^. Furthermore, as we demonstrated, the effectiveness of excitatory plasticity can be recovered after periods of adjustment, thus verifying the robustness of the artificial synapse.

In summary, the enhancing and suppressing tendencies are obtained based on the reversible charge trapping and detrapping processes in the organic semiconductor polycrystalline samples with abundant traps. The smooth carrier process can avoid the formation of conductive filament to achieve the gradually changing and quasi-continuous characteristics of conductive states for effective neural regulations. Based on this, the typical synaptic excitation, inhibition, memory formation and regression in the framework of Hebbian rules are implemented in the CuPc-based artificial synapse. Notably, the long-term homeostasis of synaptic activities can be obtained under coordinative inputs and uncoordinated inputs after self-regulation corresponding to the neural homeostatic plasticity. In another scenario of the homeostasis performance neglected by the Hebbian rules, the coupled habituation and sensitization are emulated by the activity-dependent relaxing and restoring current trends in thicker samples. These long-term memristive behaviours encourage us to exploit their connotations of dynamic balance in realistic neural networks beyond Hebbian rules. We are provided with versatile prototypes to advance simulating neuroplasticity comprehensively and further narrow the gap between biological and digital computation without intentional hard-wired connections. The CuPc samples further enlightens us to detailedly investigate the similar adaptive performances underlie the other reported architectures.

## Methods

### Samples

The sandwich device ITO/CuPc/Al is fabricated in a high vacuum chamber. After successively cleaning by acetone, ethanol, and deionized water, the glass substrate with ITO strip electrodes is treated in an ultraviolet (UV)-ozone environment for ~10 min before baking at 120 °C for ~1 h. Then the CuPc layer is gradually evaporated onto the ITO substrate in a vacuum (vacuum degree: ~5 × 10^−4^ Pa). At last, the crossed Al strip electrodes are thermally evaporated onto the CuPc layer through a shadow mask as the top electrodes (thickness: ~100 nm). For the devices with MoO_3_ buffer layer, the MoO_3_ layer is evaporated onto the ITO substrate before the CuPc layer (the thickness of the MoO_3_ is ~10 nm). The thickness of each evaporated layer is monitored by a quartz crystal cymometer in real time inside the vacuum chamber, and the changing frequency Δf corresponds to the thickness. In consideration of the CuPc layer with polydomain structure and fluctuant profile, the changing precise frequency Δ*f* is utilized to mark the evaporated film with different thickness. And there exist different conversion relationships for different materials. The thickness of prepared layers mentioned above is also measured by the step profiler.

### Measurements

The I–V characteristics of the two-terminal devices are measured by an Agilent B1500A semiconductor parameter analyzer equipped with our programming testing software in ambient condition. The devised voltage signals for specific learning rules are applied to ITO electrode and Al electrode is grounded.

## Additional Information

**How to cite this article**: Wang, L. *et al.* Long-Term Homeostatic Properties Complementary to Hebbian Rules in CuPc-Based Multifunctional Memristor. *Sci. Rep.* **6**, 35273; doi: 10.1038/srep35273 (2016).

## Supplementary Material

Supplementary Information
