# Supplementary material for: Long-Term Homeostatic Properties Complementary to Hebbian Rules in CuPc-Based Multifunctional Memristor
Source: Sci Rep. 2016 Oct 20;6:35273. doi: 10.1038/srep35273 (PMC5071877; doi:10.1038/srep35273)
Supplement: Supplementary Information [file srep35273-s1.pdf]

# Supporting Information

## Long-Term Homeostatic Properties Complementary to Hebbian Rules in CuPc-Based Multifunctional Memristor

Laiyuan Wang[1], Zhiyong Wang[1], Jinyi Lin[1,2], Jie Yang[1], Linghai Xie[1]*, Mingdong Yi[1]*, Wen Li[1], Haifeng Ling[1], Changjin Ou[2] & Wei Huang[1,2]*

### 1. CuPc polycrystalline film

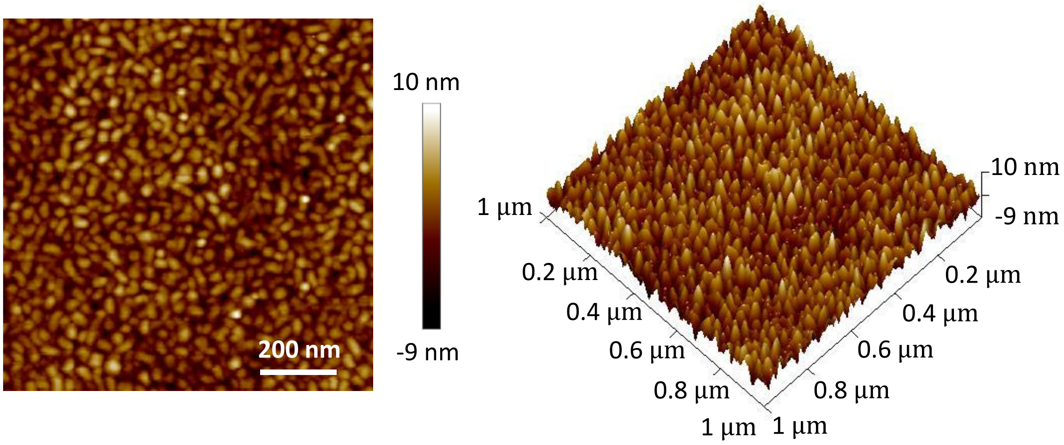


**Figure S1**. 2D (left) and 3D (right) AFM images of the organic semiconductor polycrystalline film.

## 2. The device area-dependent memristive behaviors

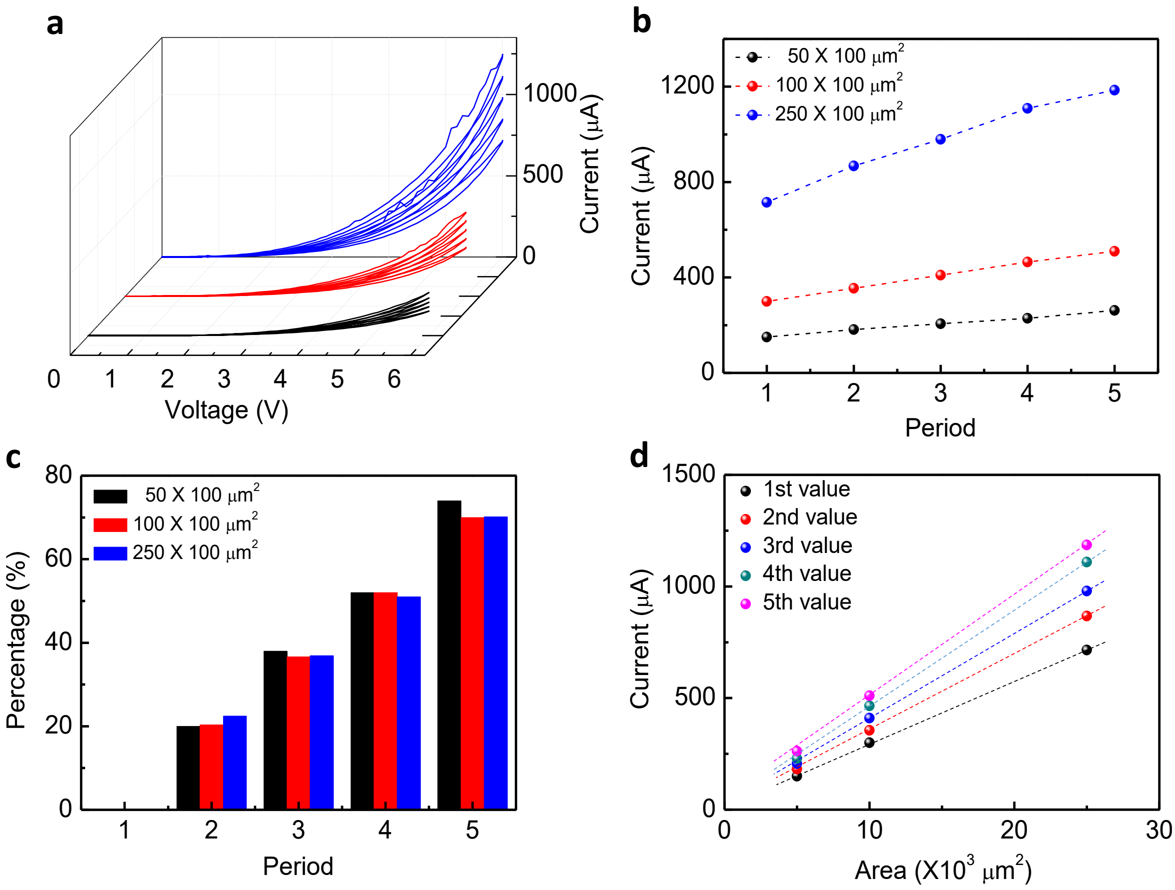


**Figure S2**. The device area-dependent memristive behaviors. (**a**) The periodic sweeping current during 0 → 6 V → 0 in three types of devices ITO/$MoO_3$/CuPc/Al with different area (50×100 μm² black curves, 100×100 μm² red curves, 250×100 μm² blue curves). The thickness of CuPc layer is 800 Hz. (**b**) The ultimate values of each cyclic sweep versus the period number extracted from **a**. (**c**) The changing percentage of the ultimate values relative to the first value ($(I_n - I_1)/I_1$, $I_n$ represents the *n*th ultimate values in **b**). The changing ultimate values and changing percentage are extracted to characterize the changing overall current. (**d**) All of the ultimate values in different area devices extracted from **b**.

## 3. PTP behaviors in CuPc-based memristor

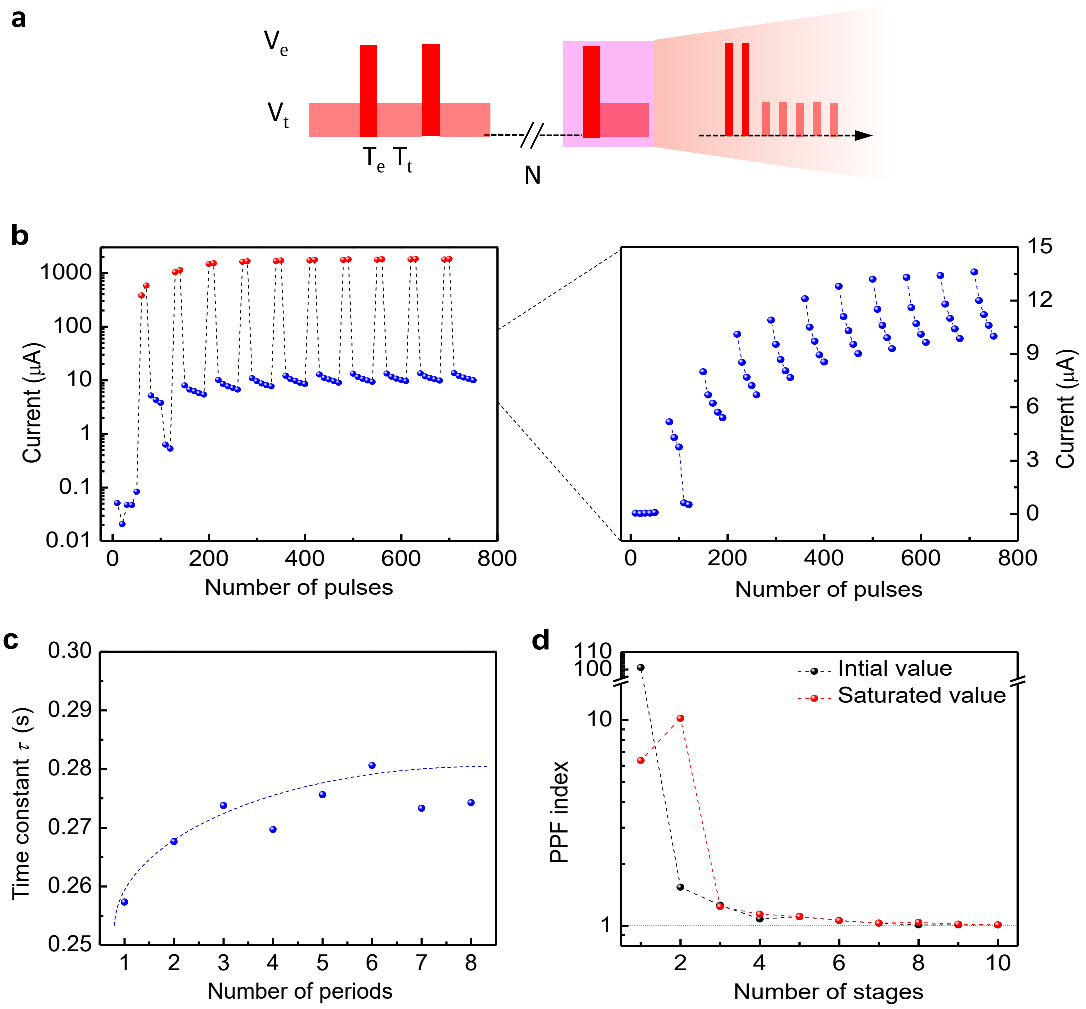


**Figure S3. PTP behaviors**. (**a**) The testing bias profile of PTP mode applied to the memristor, consisting of activated bias $V_e$ = 10 V and read bias $V_t$ = 3 V. (**b**) The overall conductive level is gradually enhanced by 10 periodic electric actions and spontaneously relaxes under testing pulses. (**c**, **d**) The relaxed time constant $\tau$ **c** and PTP index (initial one and saturated one) **d** of the relaxed stages extracted from **b** except for the first stage.

Synaptic weight and EPSC can be affected by spiking amplitude and interval. Post-tetanic-potentiation (PTP) means a serial of high-frequency actions with enough short interval can trigger remarkable increasing weight for temporal enhancement of synaptic connection in biological system, known as synaptic facilitation[S1-3]. Based on the history-dependent characteristics of conductive state in CuPc based memristor, we simulate this biological phenomenon. As Fig. S3b shows, the device is at low conductance state initially indicated by

the measured EPSC under low voltage, and the EPSC increases gradually as more active pulses are applied as explianed in Fig. S3a. Then, it fades exponentially as soon as the actions are removed, which can be fitted by the stretched exponential function. However, it fades slower implied by the increasing $\tau$ (Fig. S3c). The higher conductance and longer $\tau$ of relaxing current demonstrate the gradual enhancement of synaptic connection as more repeated actions are applied.

To investigate the PTP indexes (initial PTP index and saturated PTP index), here we define initial PTP index as the ratio between the latter initial value $A_{i,n+1}$ and former one $A_{i,n}$ in each period and saturated PTP index as ratio between the latter saturated value $A_{s,n+1}$ and former one $A_{s,n}$. Both these two indexes gradually decrease with a tendency to reach 1 (Fig. S3d), indicating that the excited initial capacity and stable capacity of EPSC tend to the saturated state. As pointed out, they are essential evidences of the transition from short-term potentiation to long-term potentiation.

## 4. STM-to-LTM transition

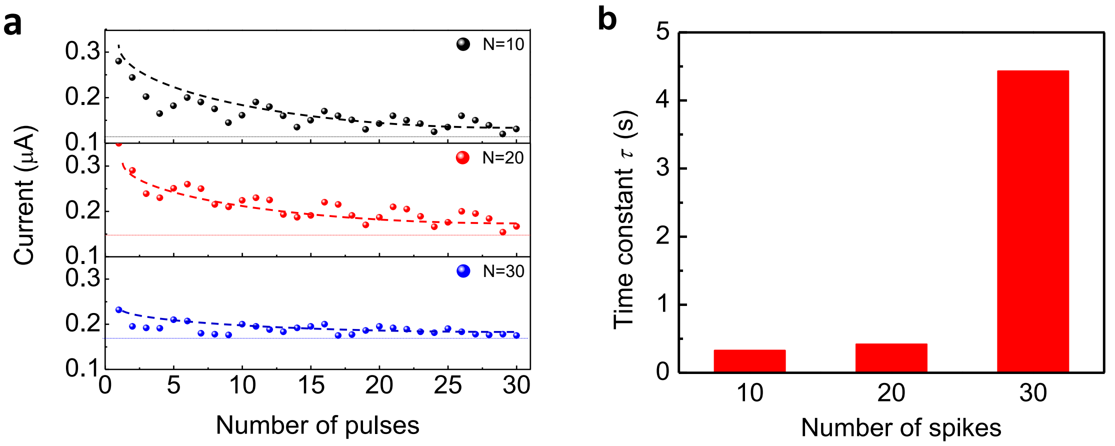


**Figure S4. STM-to-LTM transition.** (**a, b**) Relaxed behaviors of the resultant current recorded under 3 V after 10, 20, 30 identical stimuli of 10 V **a**. The overall changing trends are fitted by the stretched exponential equations, of which $\tau$ is plotted versus the number of stimuli in **b**. The dash lines indicate the relaxing saturated current.

From the relaxed curves after applying 10, 20, 30 activations (Fig. S4a), it can be conclude that longer $\tau$ is taken to relax (Fig. S4b), indicating the enhanced memory through longer time learning.

## 5. Balanced memristive responses under much more coordinate stimuli

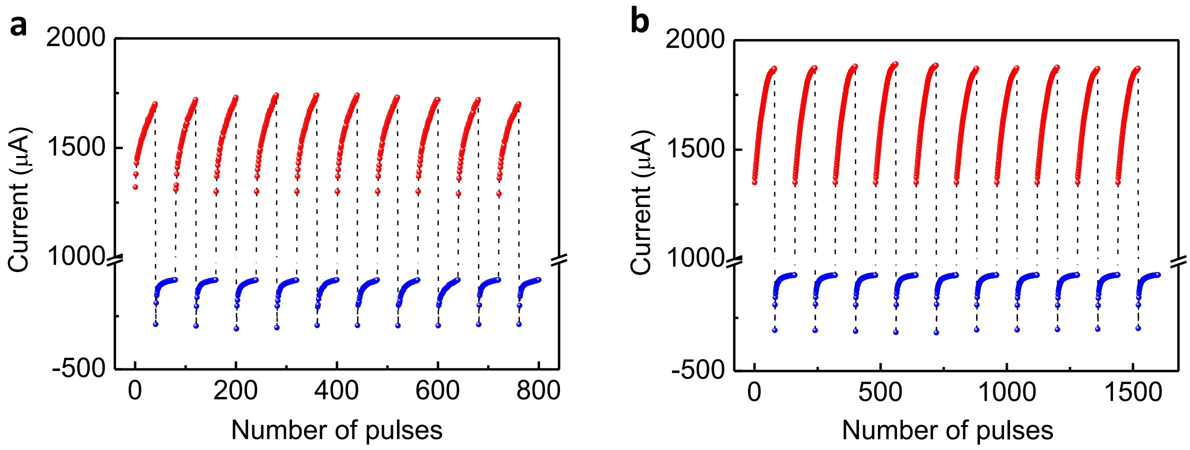


**Figure S5. Balanced memristive responses under much more coordinate stimuli.** (**a**, **b**) The periodically increasing and decreasing current under alternant positive and negative pulses of $V_e/V_i$ = 10/-10 V ($N_e/N_i$ = 40 **a**, $N_e/N_i$ = 80 **b**, N = 10 **a**, **b**).

## 6. Homeostatic plasticity under much more uncoordinate stimuli

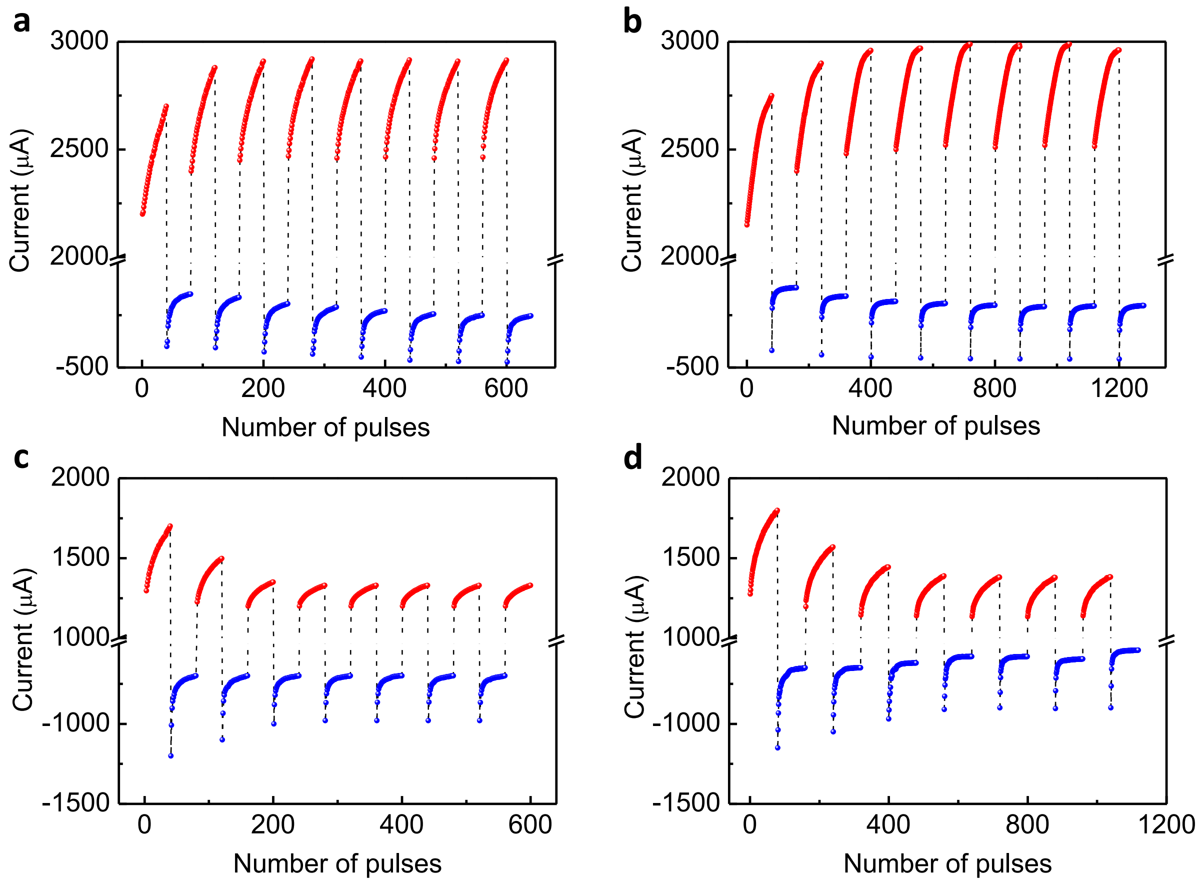


**Figure S6. Homeostatic responses under much more uncoordinate stimuli.** (**a-d**) The current gradually increases/decreases under continuous pulses of $V_e/V_i$ = 12/-10 V **a, b,** and $V_e/V_i$ = 9/-14 V **c, d** ($N_e/N_i$ = 40 **a**, **c**, $N_e/N_i$ = 80 **b**, **d**, N = 10 **a-d**). (**a, b**) Compared with the response modes under coordinated stimuli in Fig. S5, the positive current level grows stronger, while the overall negative current is gradually enhanced till they reach a homeostatic state as more periods are applied. (**c, d**) The overall positive and negative current levels are gradually depressed till they reach a new balanced state.

### 7. Pulse number-dependent amplifying variations of the overall activities relative to the initial states

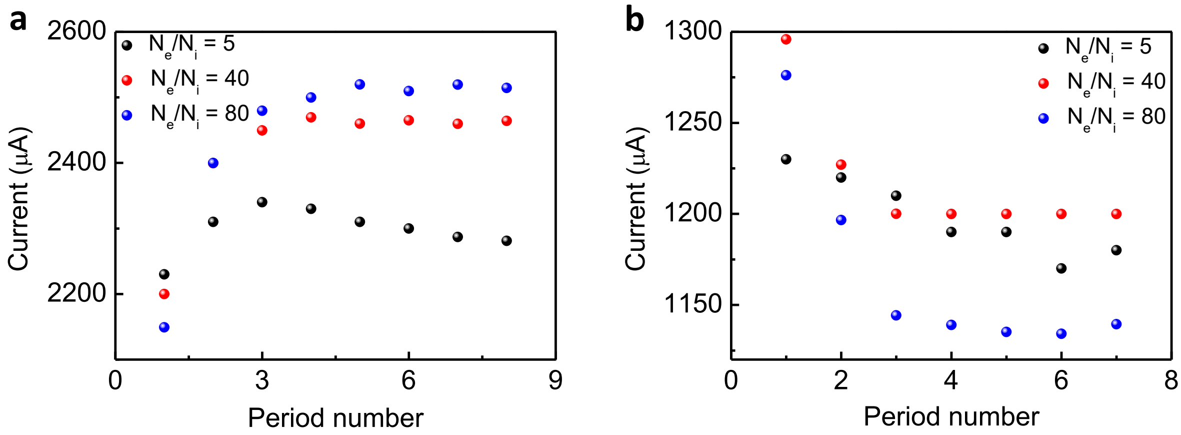


**Figure S7. (a, b) The initial values of each period to reflect the gradually adaptive device state of different modes with increasing $N_e/N_i$** ($V_e/V_i$ = 12/-10 V **a**, $V_e/V_i$ = 9/-14 V **b**, the values of $N_e/N_i$ = 5 mode are extracted from Fig. 4 in the manuscript and the values of $N_e/N_i$ = 40, 80 modes are extracted from Fig. S6 above).

## 8. Memristive characteristics in the thicker CuPc samples

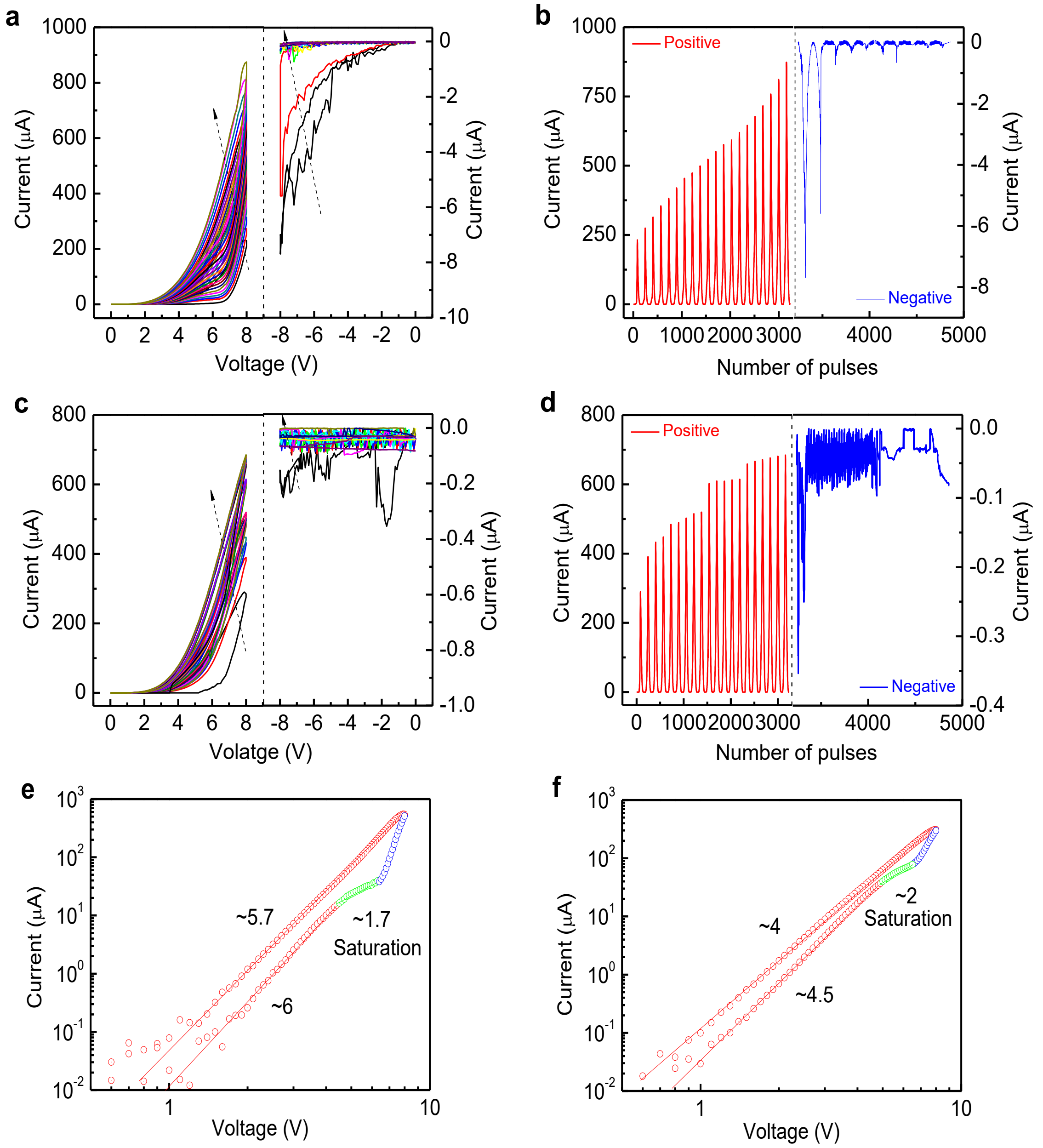


**Figure S8. Tunable conductance under cyclic sweeps of thicker CuPc-based memristors.** (**a, c**) Cyclic I-V characteristics under consecutive positive/negative voltage sweeps 0 → 8/-8 V → 0 in ITO/$MoO_3$/CuPc (1600 Hz)/Al **a** and ITO/$MoO_3$/CuPc (3200 Hz)/Al **c**. (**b, d**) The changing trend of current versus pulse number extracted from data in **a, c** respectively. (**e, f**) Log-log plots of current during the first cyclic sweeps in thicker memristors extracted from **a** and **c**.

The hysteresis loops of current under positive/negative voltage sweeps likewise gradually

increase/decrease in thicker CuPc memristors. However, it can be noted that there are two main differences compared with aforementioned thin device as presented in Fig. 2 and Fig. S8a, c. As the thickness is thickened, the conductive levels are suppressed, especially the negative level, and the rectification ratio is enlarged.

## 9. Periodic testing mode for the emulations of habituation and sensitization

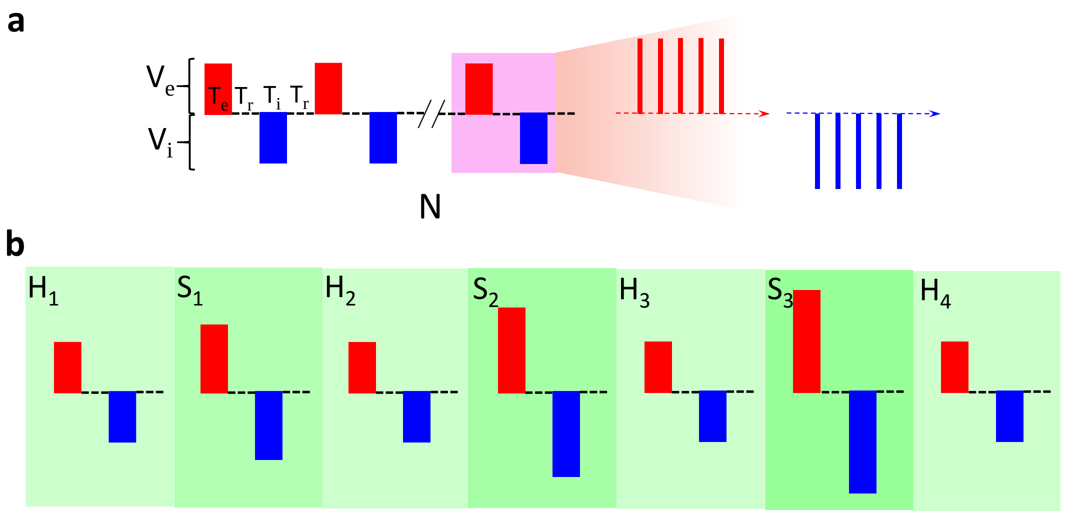


**Figure S9. (a)** Shematic diagram of the alternant bias mode for the emulations of habituation and sensitization in thicker CuPc devices including excitatory stage $T_e$, resting stage $T_r$, inhibitory stage $T_i$ and resting stage $T_r$ successively in each period ($T_e = T_i = T_r$, 5 pulse in each stage). N is the number of applied periods. **(b)** The pulsing schemes applied in Fig. 6 are $H_1$: $V_e/V_i$ = 6/-6 V, N = 18 **a;** $S_1$: $V_e/V_i$ = 8/-8 V, N = 6 **b;** $H_2$: $V_e/V_i$ = 6/-6 V, N = 18 **c;** $S_2$: $V_e/V_i$ = 10/-10 V, N = 6 **d;** $H_3$: $V_e/V_i$ = 6/-6 V, N = 18 **e;** $S_3$: $V_e/V_i$ = 12/-12 V, N = 6 **f;** $H_4$: $V_e/V_i$ = 6/-6 V, N = 4 **g.** $V_e/V_i$ = 10/-10 V, N = 18 **h.**

## 10. The recovered habitualized current levels

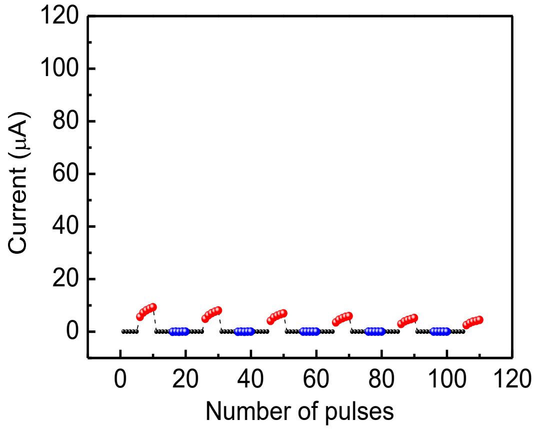


**Figure S10.** The habitualized current levels can be nearly recovered after resting 3 hours in the thicker device.

**Supplementary References**


S1. Gingrich K. J. & Byrne J. H. Simulation of synaptic depression, posttetanic potentiation, and presynaptic facilitation of synaptic potentials from sensory neurons mediating gill-withdrawal reflex in Aplysia. *J. Neurophysiol.* **53**, 652-669 (1985).

S2. Felmy F., Neher E. & Schneggenburger R. Probing the intracellular calcium sensitivity of transmitter release during synaptic facilitation. *Neuron* **37**, 801-811 (2003).

S3. Ghirardi M., Montarolo P. G. & Kandel E. R. A novel intermediate stage in the transition between short-and long-term facilitation in the sensory to motor neuron synapse of Aplysia. *Neuron* **14**, 413-420 (1995).
